# Vascular adhesion protein-1-targeted PET imaging in autoimmune myocarditis

**DOI:** 10.1007/s12350-023-03371-8

**Published:** 2023-09-27

**Authors:** Arghavan Jahandideh, Jenni Virta, Xiang-Guo Li, Heidi Liljenbäck, Olli Moisio, Jesse Ponkamo, Noora Rajala, Marion Alix, Jukka Lehtonen, Mikko I. Mäyränpää, Tiina A. Salminen, Juhani Knuuti, Sirpa Jalkanen, Antti Saraste, Anne Roivainen

**Affiliations:** 1 Turku PET Centre, University of Turku, Åbo Akademi University and Turku University Hospital, 20520 Turku, Finland; 2 InFLAMES Research Flagship Center, University of Turku, Turku, Finland; 3 Department of Chemistry, University of Turku, Turku, Finland; 4 Turku Center for Disease Modeling, University of Turku, Turku, Finland; 5 Structural Bioinformatics Laboratory, Åbo Akademi University, Turku, Finland; 6 Heart and Lung Center, Helsinki University and Helsinki University Hospital, Helsinki, Finland; 7 Department of Pathology, Helsinki University and Helsinki University Hospital, Helsinki, Finland; 8 MediCity Research Laboratory and Institute of Biomedicine, University of Turku, Turku, Finland; 9 Heart Center, Turku University Hospital and University of Turku, Turku, Finland

**Keywords:** Experimental autoimmune myocarditis, myocarditis, positron emission tomography, sarcoidosis, Siglec-9, vascular adhesion protein-1

## Abstract

**Background:**

Vascular adhesion protein-1 (VAP-1) is an adhesion molecule and primary amine oxidase, and Gallium-68-labeled 1,4,7,10-tetraazacyclododecane-*N*,*N*′,*N*″,*N*‴-tetra-acetic acid conjugated sialic acid-binding immunoglobulin-like lectin 9 motif containing peptide ([^68^Ga]Ga-DOTA-Siglec-9) is a positron emission tomography (PET) tracer targeting VAP-1. We evaluated the feasibility of PET imaging with [^68^Ga]Ga-DOTA-Siglec-9 for the detection of myocardial lesions in rats with autoimmune myocarditis.

**Methods:**

Rats (n = 9) were immunized twice with porcine cardiac myosin in complete Freund’s adjuvant. Control rats (n = 6) were injected with Freund’s adjuvant alone. On day 21, in vivo PET/computed tomography (CT) imaging with [^68^Ga]Ga-DOTA-Siglec-9 was performed, followed by ex vivo autoradiography, histology, and immunohistochemistry of tissue sections. In addition, myocardial samples from three patients with cardiac sarcoidosis were studied.

**Results:**

[^68^Ga]Ga-DOTA-Siglec-9 PET/CT images of immunized rats showed higher uptake in myocardial lesions than in myocardium outside lesions (SUV_mean_, 0.5 ± 0.1 *vs* 0.3 ± 0.1; *P* = .003) or control rats (SUV_mean_, 0.2 ± 0.03; *P* < .0001), which was confirmed by ex vivo autoradiography of tissue sections. Immunohistochemistry showed VAP-1-positive staining in lesions of rats with myocarditis and in patients with cardiac sarcoidosis.

**Conclusion:**

VAP-1-targeted [^68^Ga]Ga-DOTA-Siglec-9 PET is a potential novel technique for the detection of myocardial lesions.

**Supplementary Information:**

The online version contains supplementary material available at 10.1007/s12350-023-03371-8.

## Introduction

Myocarditis is an inflammatory disease of the heart characterized by infiltration of the myocardium by immune cells, myocyte necrosis, and scarring, and may be associated with ventricular arrhythmias, conduction disturbances, and inflammatory cardiomyopathy.^1^ Myocarditis may result from infection, cardiotoxic agents, or autoimmune diseases such as sarcoidosis.

Diagnosis of myocarditis is challenging; currently, endomyocardial biopsy is considered the gold standard for diagnosing myocarditis, but its sensitivity is low because of the focal nature of cardiac lesions.^2^ Thus, advanced non-invasive cardiac imaging techniques including cardiac magnetic resonance (CMR) imaging and positron emission tomography (PET) are increasingly used to characterize and define myocardial abnormalities.^3,4^ PET/computed tomography (CT) with 2-deoxy-2-[^18^F]fluoro-d-glucose ([^18^F]FDG) is a sensitive non-invasive imaging technique that has shown good accuracy in detecting myocardial inflammation.^4^ However, physiological [^18^F]FDG uptake in normal myocardium may interfere with the assessment of abnormal uptake and affect its specificity.^5^ Therefore, attempts have been made to develop more specific PET tracers for the detection of myocarditis.^6–12^

Vascular adhesion protein-1 (VAP-1) is an endothelial cell surface molecule involved in the migration of leukocytes from the blood to tissue during acute and chronic inflammation.^13^ During inflammation, VAP-1 is upregulated and rapidly translocated from intracellular storage granules to the surface of endothelial cells. A soluble form of VAP-1 (sVAP-1) derived from the membrane-bound form is normally present at low levels in serum.^14^ VAP-1 activation is associated with many inflammatory conditions, including multiple sclerosis, atherosclerosis, dermatitis, inflammatory liver disease, and arthritis,^14–17^ making VAP-1 a potential target for in vivo imaging of inflammation. Sialic acid-binding immunoglobulin-like lectins (Siglec) 9 and 10 are leukocyte ligands of VAP-1. We have previously demonstrated that Gallium-68-labeled 1,4,7,10-tetraazacyclododecane-*N*,*N*′,*N*″,*N*‴-tetra-acetic acid conjugated Siglec 9 motif containing peptide ([^68^Ga]Ga-DOTA-Siglec-9) can be utilized as a PET tracer for imaging inflammation in both disease models and in humans.^14,18–21^

In this study, we evaluated the feasibility of VAP-1-targeting [^68^Ga]Ga-DOTA-Siglec-9 imaging for the detection of myocardial inflammation in rat autoimmune myocarditis.

## Methods

### Animal model and study protocol

Autoimmune myocarditis was induced as previously described.^6^ Nine male Lewis rats aged 6 to 8 weeks (weight, 302.2 ± 17.3 g) were twice immunized with subcutaneous injections of 5 mg/mL pig cardiac myosin (M0531; Sigma Aldrich) in an equal volume of complete Freund’s adjuvant supplemented with 1 mg/mL mycobacterium tuberculosis (F5881; Sigma Aldrich). The two inoculations were given 7 days apart into the hock of the left foot. An intraperitoneal injection of 250 ng/mL pertussis toxin (P2980; Sigma Aldrich) was also given at the time of the first inoculation to enhance immunization. Six control rats (weight, 331.0 ± 9.5 g) twice received the adjuvant alone. All procedures were carried out under isoflurane anesthesia (1.5% to 2.5%). For analgesia, buprenorphine (0.03 mg/kg) was administered two times a day for 2 days after immunization.

**Figure 1:**
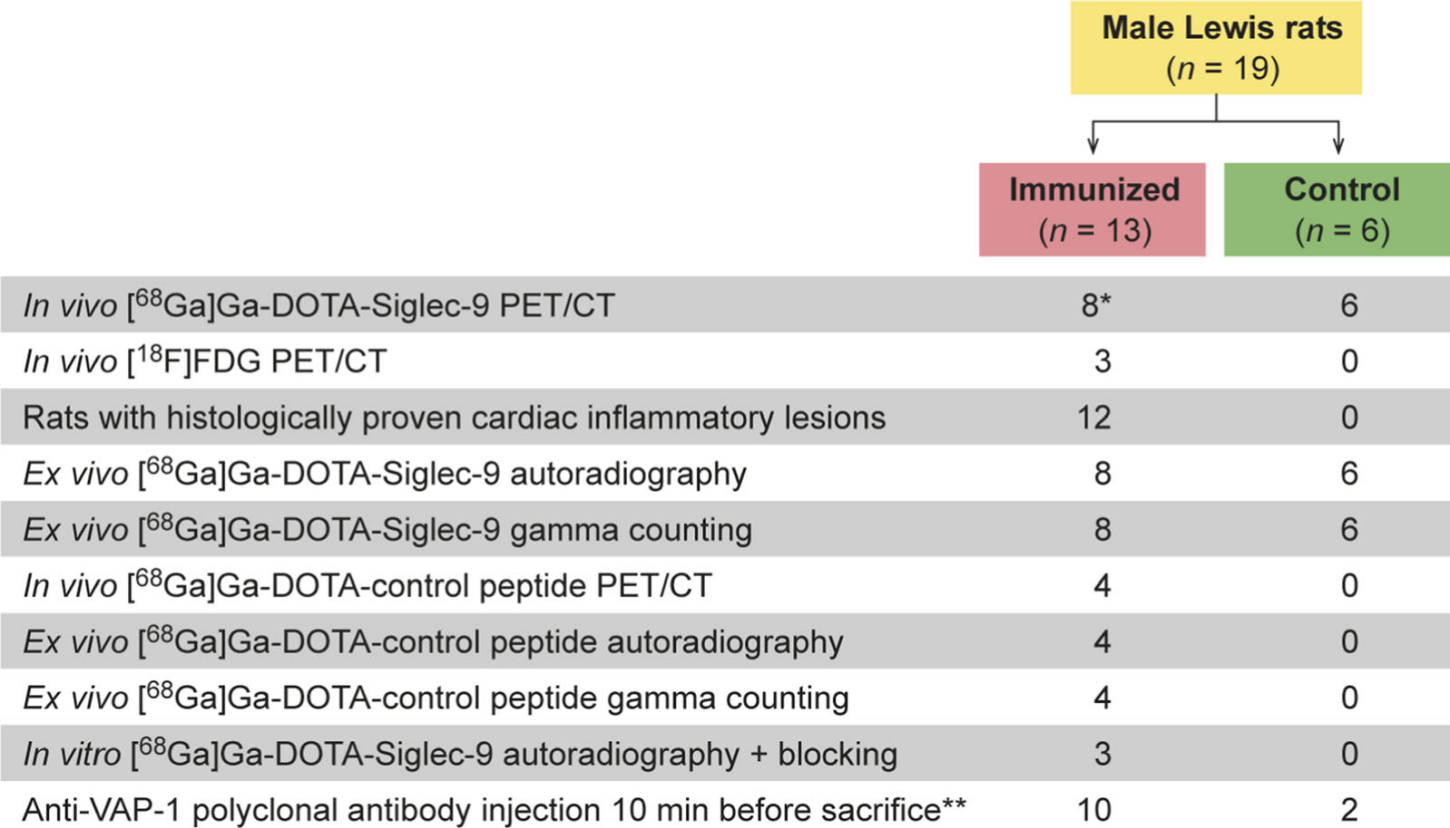
A flow diagram of the study design and the numbers of animals used. *Four had a dynamic scan and the others a static scan. **A dose of 1 mg/kg of anti-VAP-1 polyclonal antibody was injected intravenously 10 minutes before sacrifice to detect surface-bound VAP-1 by immunofluorescence

PET imaging with [^68^Ga]Ga-DOTA-Siglec-9 and contrast-enhanced CT were performed on day 21 after the first immunization. To localize the myocardium, contrast-enhanced CT was performed immediately after the PET scan. In three immunized rats, an [^18^F]FDG scan was acquired 1 day before the [^68^Ga]Ga-DOTA-Siglec-9 injection to further facilitate the localization of the myocardium. A dose of 1 mg/kg of anti-VAP-1 polyclonal antibody was injected intravenously 10 minutes before sacrifice into six immunized rats and two controls to allow detection of luminal VAP-1 by immunofluorescence. The imaged rats were euthanized at 70 minutes post-injection of [^68^Ga]Ga-DOTA-Siglec-9, blood was drawn by cardiac puncture, and the heart and other organs were excised, weighed, and measured for radioactivity to determine tracer biodistribution using a gamma counter (Triathler 3″; Hidex).^6^ The heart was embedded in optimal cutting temperature compound and frozen, and then cut into serial transverse 20 µm and 8 µm cryosections at 1 mm intervals from the base to apex for autoradiography, histology, and immunostaining. A flow diagram of the study protocol and the numbers of rats used is shown in Figure 1.

All animal experiments were approved by the national Project Authorization Board (permission number ESAVI/968/04.10.07/2018 and ESAVI/37123/2020) and were carried out in compliance with the EU Directive 2010/EU/63 on the protection of animals used for scientific purposes.

### Radiotracers

[^18^F]FDG was prepared using standard FASTLab® cassettes.^22^ [^68^Ga]Ga-DOTA-Siglec-9 was prepared according to previously described procedures.^14,18^ Briefly, a cyclic peptide, CARLSLSWRGLTLCPSK, with disulfide bridged cysteines consisting of residues 283 to 297 from the Siglec-9 and 8-amino-3,6-diooxaoctanoyl linker (polyethylene glycol derivative) between DOTA chelator and peptide (Peptide Specialty Laboratories GmbH), was labeled with ^68^Ga. The radiochemical purity determined by reversed-phase radio-HPLC was > 95% and the molar activity was 25.8 ± 8.3 GBq/µmol.

The [^68^Ga]Ga-DOTA-control peptide was created from the cyclic [^68^Ga]Ga-DOTA-Siglec-9 peptide CARLSLSWRGLTLCPSK by first mutating Arg3, Trp8, and Arg9 to alanines. These residues were selected since the double mutated R3A/R9A peptide does not bind human VAP-1 (hVAP-1).^20^ Trp8 was additionally mutated to alanine since its predicted binding site is not conserved in human VAP-1 and rat VAP-1.^23^ Thereafter, the residues in the resulting CAALSLSAAGLTLCPSK peptide were scrambled manually by avoiding Siglec-9-derived amino acid repeats and by distributing polar residues throughout the sequence to avoid hydrophobic patches. The constructed [^68^Ga]Ga-DOTA-control, CSALATGLSALALCPSK, the R3A/R9A, and the [^68^Ga]Ga-DOTA-Siglec-9 peptides were docked into the homology model for rat VAP-1 (rVAP-1) with Gold^24^ using the pyridazinone inhibitor in the crystal structure of hVAP-1 (Protein Data Bank (PDB) ID 4bty)^25^ as the center of the binding site. The rVAP-1 model was created with Modeller^26^ using the published sequence alignment^23^ and hVAP-1 structure (PDB ID 4bty). Additionally, all peptides were docked into hVAP-1 for comparison. Docking of the peptides to hVAP-1 and rVAP-1 resulted in 10 binding modes for each peptide. Trp8 in the [^68^Ga]Ga-DOTA-Siglec-9 peptide binds close to the specificity pocket of hVAP-1 (Asp180, Thr210, and Leu177).^23,25^ This binding site is not conserved in rVAP-1 where the corresponding residues are Gln180, Lys210, and Gln177.^23^ Trp8 in the docked [^68^Ga]Ga-DOTA-Siglec-9 and R3A/R9A peptides binds near Gln180 and Gln177 in rVAP-1, whereas the constructed ^68^Ga-DOTA-control with scrambled sequence and without any tryptophanes does not interact with these residues (Supplemental Figure 1).

### In vivo PET/CT imaging

A small animal PET/CT scanner (Inveon Multimodality; Siemens Medical Solutions) was used to perform in vivo imaging with [^68^Ga]Ga-DOTA-Siglec-9 and [^18^F]FDG in the rats. The rats were injected with 50.4 ± 2.3 MBq [^68^Ga]Ga-DOTA-Siglec-9 via the tail vein. Sixty-minute dynamic PET acquisitions (6 × 10 seconds, 4 × 60 seconds, 5 × 300 seconds, and 3 × 600 seconds time frames) were acquired from four immunized rats to study the kinetics of the tracer. Thirty-minute static PET starting at 30 minutes post-injection was then acquired from the rest of the rats. The PET data were reconstructed using an iterative three-dimensional ordered subset expectation maximization using maximum a priori with shifted Poisson distribution (OSEM3D/SP-MAP) algorithm with 2 OSEM iterations and 18 MAP iterations, attenuation and dead time correction, target resolution 1.5 mm, and matrix size 128 × 128.

To visualize myocardium, a 40 minute static [^18^F]FDG (40.4 ± 1.6 MBq) PET acquisition starting at 20 minutes post-injection was performed the day before the [^68^Ga]Ga-DOTA-Siglec-9 study in three immunized rats. In addition, 300 μL of intravascular iodinated contrast agent (eXia™ 160XL; Binitio Biomedical Inc.) was injected into all rats to acquire high-resolution CT imaging immediately after the [^68^Ga]Ga-DOTA-Siglec-9 PET, as described in a previous study.^18^

Uniformly sized regions of interest (ROIs) were defined in the left ventricle (LV) myocardium in the [^68^Ga]Ga-DOTA-Siglec-9 images, which were co-registered with the CT or [^18^F]FDG uptake maps using Carimas 2.9 software (Turku PET Centre). These ROIs were based on macroscopically defined inflammation in the hematoxylin and eosin (H&E) stained sections, with or without visually increased in vivo [^68^Ga]Ga-DOTA-Siglec-9 uptake. ROIs were also defined in the septal wall to measure tracer uptake in the myocardium outside lesions. The mean standardized uptake value (SUV_mean_) was compared between histologically confirmed myocardial lesions and myocardium outside lesions. Additional ROIs were defined in the lung, liver, kidney, foreleg muscle, LV cavity, and vena cava (blood). In rats for which dynamic datasets were acquired, decay-corrected time-activity curves and myocardial lesion-to-blood (average of LV and vena cava) uptake ratio curves were determined.

### Ex vivo autoradiography, histology, and immunostaining

Ex vivo digital autoradiography was performed on 20 μm cryosections of the myocardium of the imaged rats with [^68^Ga]Ga-DOTA-Siglec-9, as previously described.^14,18^ ROIs were defined in myocardial lesions and myocardium outside lesions on the basis of H&E staining of the same section, and the results are presented as average photo-stimulated luminescence per square millimeter (PSL/mm^2^).

For general histology, H&E staining was performed on 20 μm cryosections. For immunohistochemistry, 8 μm cryosections were stained with a monoclonal mouse anti-rat CD68 antibody (1:1000, MCA341GA, Bio-Rad) to detect macrophages, a polyclonal CD31 antibody (1:200, NB100-2284, Novus Biologicals) to detect vascular endothelium, and monoclonal mouse anti-actin α-smooth muscle antibody (1:12,000, A5228-200, Sigma Aldrich) to detect α-smooth muscle actin (α-SMA) in myofibroblasts with appropriate peroxidase conjugated second stage antibodies. 3,3′-Diaminobenzidine (DAB) was used as a chromogen. Immunofluorescence staining with anti-VAP-1 antibody was performed on 8 μm cryosections to detect VAP-1, as previously described.^14^ The heart cryosections of the rats that received intravenous anti-VAP-1 antibody (clone 174-5, 1 mg/kg diluted in saline) 10 minutes before sacrifice (five immunized and two controls) were incubated with fluorescein isothiocyanate (FITC)-conjugated anti-mouse IgG (1:100, Sigma F28883) + 5% normal rat serum followed by Alexa Fluor® 488 conjugated anti-FITC (5 mg/mL Invitrogen A11029) and Hoechst (1:5000, Thermo Scientific 62249). The heart cryosections of the other rats (four immunized and four controls) were incubated with polyclonal anti-VAP-1 antibody,^14^ followed by Alexa Fluor® 488 conjugated goat anti-rabbit IgG secondary antibody (Life Technologies, A11034).

Additional H&E and immunohistochemical staining with a monoclonal mouse anti-rat CD68 antibody (1:2000, MCA341GA, Bio-Rad) and a polyclonal anti-VAP-1 antibody^14^ were performed on 4 µm paraffin-embedded sections and 8 μm cryosections of thymus, white adipose tissue, spleen, and bone marrow of immunized rats that received intravenous anti-VAP-1 antibody (clone 174-5, 1 mg/kg diluted in saline) 10 minutes before sacrifice.

### Human myocardial samples

Two myocardial autopsy samples from two patients who died from cardiac sarcoidosis and one myocardial sample of a heart explanted because of sarcoidosis were cut into serial 4-μm paraffin sections. Use of human samples was approved by hospital ethical review board (HUS317/13/03/01/2015 and HUS/1068/2016), the National Authority for Medicolegal Affairs (4615/06.01.03.01/2016), and the National Institute for Health and Welfare (THL/691/5.05.00/2016).^27^

H&E staining was performed for general histology. Monoclonal mouse anti-human CD68 antibody (1:200, M0876, Dako Agilent Technologies Inc.) and polyclonal anti-VAP-1 antibody produced in rabbits against recombinant human VAP-1^28^ were used to detect macrophages and VAP-1, respectively, in myocardial lesions.

### Studies with [^68^Ga]Ga-DOTA-control peptide

An in vivo assessment of [^68^Ga]Ga-DOTA-control peptide binding was performed in four immunized rats (weight, 298.7 ± 9.5 g). The rats were injected with 40.8 ± 5.8 MBq [^68^Ga]Ga-DOTA-control peptide via the tail vein and were euthanized at 70 minutes post-injection, after which biodistribution analysis and ex vivo autoradiography were performed on 20 μm cryosections of the myocardium.

### In vitro binding of [^68^Ga]Ga-DOTA-Siglec-9

To further evaluate the specificity of the tracer, in vitro binding of [^68^Ga]Ga-DOTA-Siglec-9 in myocardial tissue sections from three immunized rats was studied with or without 10 minutes pre-incubation with a 100-fold molar excess of non-labeled DOTA-Siglec-9 peptide as a blocker. Total and blocked binding of [^68^Ga]Ga-DOTA-Siglec-9 was assessed by digital autoradiography.

### Statistical analysis

Data are presented as mean ± standard deviation (SD). Unpaired or paired *t* tests were applied to test single comparisons between normally distributed data to evaluate differences in [^68^Ga]Ga-DOTA-Siglec-9 uptake in healthy myocardium (control rats), myocardial lesions, and myocardium outside lesions. Mann–Whitney *U* test and Kolmogorov–Smirnov test were applied to single comparisons between non-normally distributed data. Normality distribution assumption was checked with Shapiro–Wilk test, and assumption of variance equality was checked with Fisher’s *F* test. GraphPad Prism 6 Software was used to perform the analyses, and statistical significance was set at *P* < .05.

## Results

### VAP-1 in rat autoimmune myocarditis

**Figure 2:**
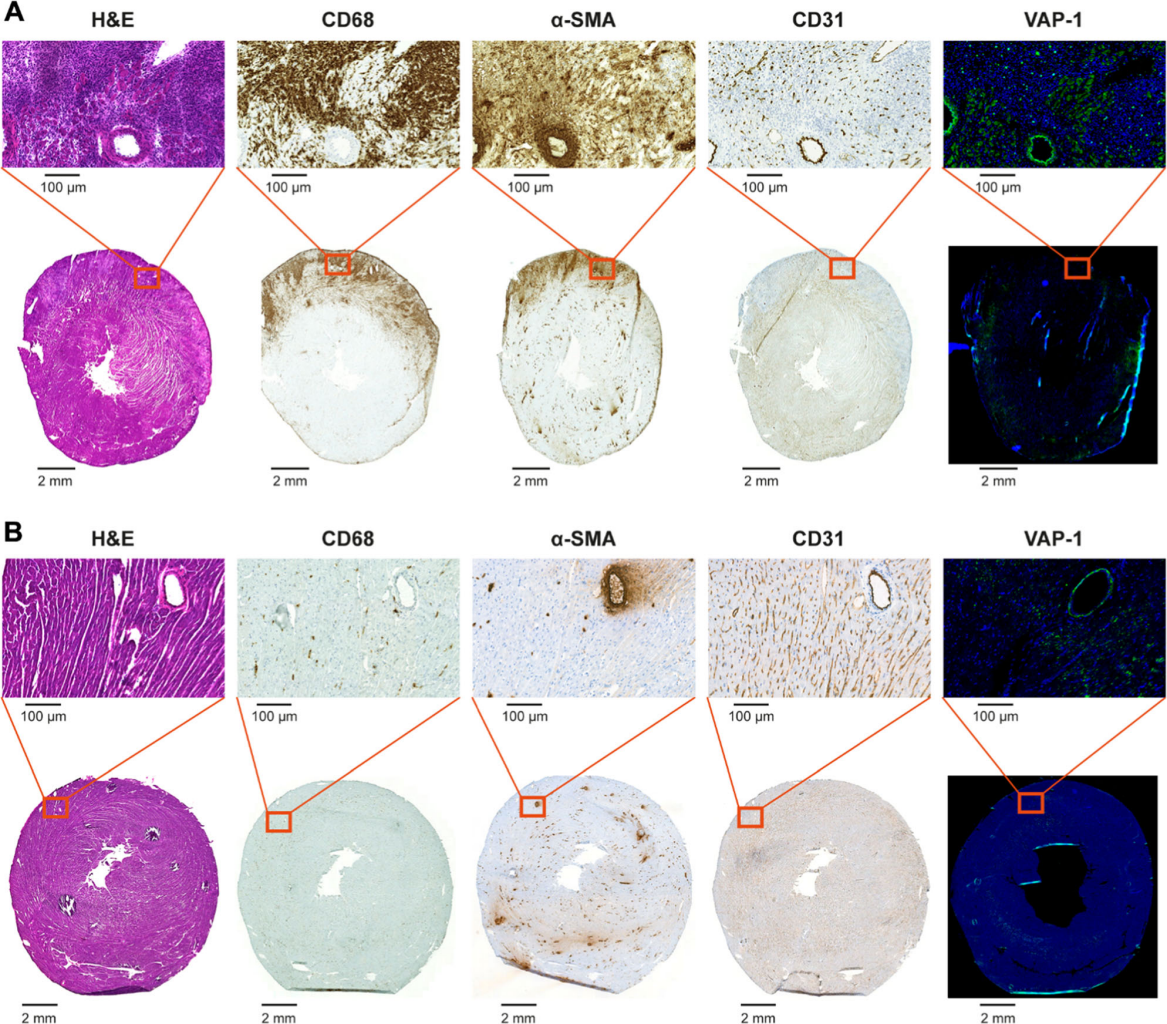
(**A**) A myocardial lesion from a rat with autoimmune myocarditis identified by hematoxylin and eosin (H&E) staining. A consecutive cryosection stained with immunofluorescence shows intracellular and surface-bound vascular adhesion protein-1 (VAP-1). Rat received intravenous anti-VAP-1 antibody 10 minutes before sacrifice and staining was performed using only the secondary antibody. There are numerous CD68-positive macrophages in the inflammatory lesion as well as α-smooth muscle actin (α-SMA) on myofibroblasts. Staining with antibody against CD31 indicates the presence of vascular endothelial cells. (**B**) Histology and immunohistochemical and immunofluorescence staining of a control rat myocardium

Representative histological, immunohistochemical, and immunofluorescence stainings of rat myocardium 21 days after the first immunization are shown in Figure 2A. Multiple myocardial lesions were histologically proven in eight (88%) out of nine immunized rats, whereas no lesions were detected in the six control rats (Figure 2B).

The myocardial lesions showed intense inflammatory cell infiltration, myocyte necrosis, and scarring. Consistent with previous findings,^6^ CD68-positive macrophages were the principal cells in the lesions. Immunofluorescence staining after either intravenous injection or incubation of tissue sections with anti-VAP-1 antibody revealed moderate VAP-1-positive staining at the site of lesions, whereas only occasional VAP-1 positivity was seen in the myocardium outside lesions of immunized rats and in the myocardium of control rats (Figure 2). Immunohistochemistry showed abundant α-SMA-positive cells in the myocardial lesions, whereas only occasional α-SMA-positive cells were detected in the myocardium outside lesions of immunized rats or the myocardium of control rats. CD31 staining showed the presence of capillary networks within the myocardial lesions and a proportion of CD31-positive endothelial cells.

### VAP-1 in human cardiac sarcoidosis samples

**Figure 3:**
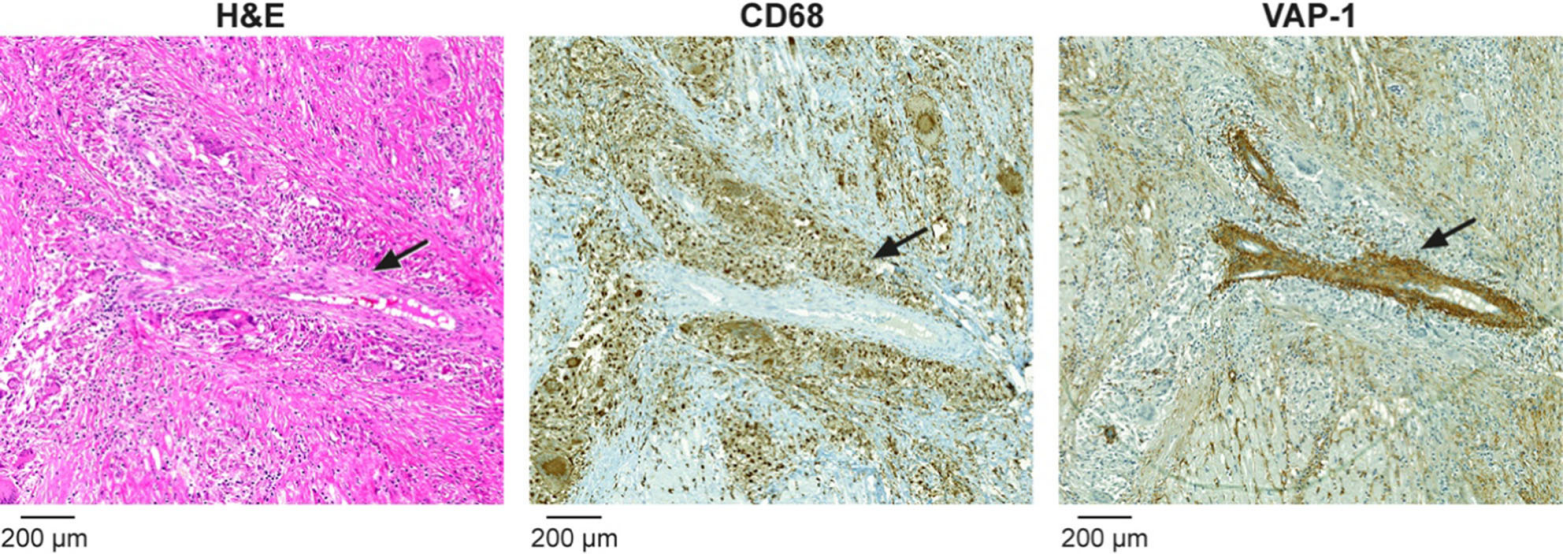
Vascular adhesion protein-1 (VAP-1) is detected in a human coronary artery smooth muscle cells and in the endothelial cells of capillaries. Black arrow in hematoxylin and eosin (H&E) staining indicates the artery inside a sarcoid granuloma. Granuloma forming giant cells and macrophages around the artery (black arrow) are highlighted in CD68-staining. VAP-1 staining of consecutive section shows intensive VAP-1-staining in arterial medial smooth muscle cells and less intense staining in the capillary endothelial cells

All human samples from patients with cardiac sarcoidosis (n = 3) showed VAP-1-positivity in myocardial lesions, which was co-localized with vascular structures (Figure 3, Supplemental Figure 2). Occasional VAP-1 positivity was also seen in the myocardium outside lesions of the patients with cardiac sarcoidosis.

### In vivo PET/CT imaging

**Figure 4:**
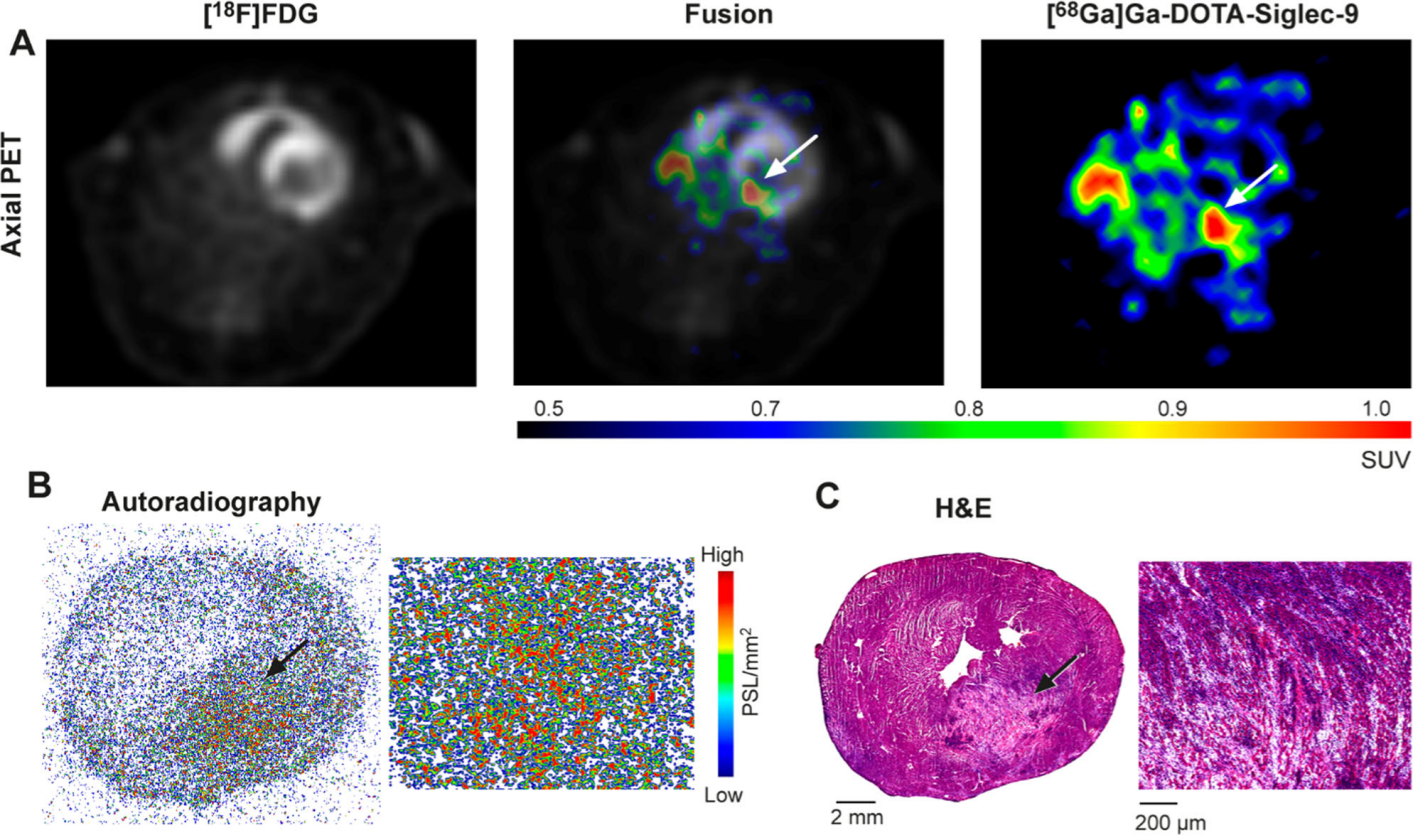
(**A**) Representative in vivo PET images with [^18^F]FDG and [^68^Ga]Ga-DOTA-Siglec-9 tracers in a rat with autoimmune myocarditis. PET images at 30 to 60 minutes show focal [^68^Ga]Ga-DOTA-Siglec-9 uptake (white arrows) in the posterior left ventricle (LV) wall. (**B**) Ex vivo autoradiography of [^68^Ga]Ga-DOTA-Siglec-9 uptake shows co-localization with edematous and hemorrhagic myocardial lesion in the posterior wall of the LV, (**C**) which is confirmed by consecutive sections stained with hematoxylin and eosin (H&E)

In vivo PET/CT imaging with [^68^Ga]Ga-DOTA-Siglec-9 allowed visualization of histologically proven myocardial lesions in all eight immunized rats (Figure 4A). Myocardial tracer uptake was not visible in the immunized rat without histological lesions, nor in the control rats (n = 6).

**Figure 5:**
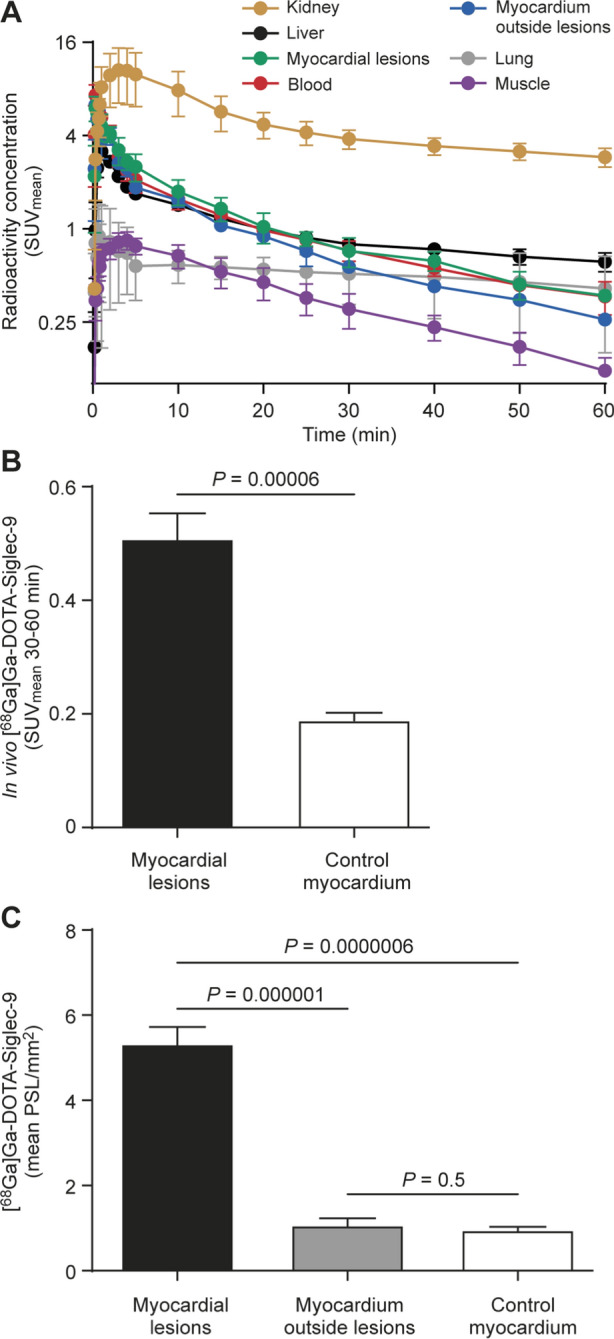
Quantification of [^68^Ga]Ga-DOTA-Siglec-9 PET imaging. (**A**) Time-activity curves from rats with autoimmune myocarditis (n = 4) show that [^68^Ga]Ga-DOTA-Siglec-9 uptake (mean standardized uptake value, SUV_mean_) remains slightly higher in the myocardial lesions than in the blood (average of inferior vena cava and left ventricle). Bars indicate standard deviation. Note that the *y*-axis has a logarithmic scale. (**B**) The average myocardial [^68^Ga]Ga-DOTA-Siglec-9 uptake (SUV_mean_) 30 to 60 minutes after injection is higher in the myocardium of immunized rats than in the myocardium of control rats (n = 6). (**C**) Bars demonstrate [^68^Ga]Ga-DOTA-Siglec-9 uptake by ex vivo autoradiography as mean photo-stimulated luminescence per square millimeter (PSL/mm^2^) in myocardial lesions and myocardium outside lesions of immunized rats (n = 8), and myocardium of control rats (n = 6). Values are mean ± SD; unpaired *t* test for comparison of immunized rats’ myocardium and control myocardium, and paired *t* test for comparison of myocardial lesions and myocardium outside lesions

[^68^Ga]Ga-DOTA-Siglec-9 radioactivity was rapidly cleared from the blood, as demonstrated by time-activity curves, while the uptake in myocardial lesions was discernible from the blood at 30 minutes post-injection (Figure 5A). The highest uptake was seen in the kidneys (Figure 5A).

[^68^Ga]Ga-DOTA-Siglec-9 uptake at 30 to 60 minutes after injection in the myocardial lesions was higher than the uptake in the control rats’ myocardium (SUV_mean_, 0.5 ± 0.1 *vs* 0.2 ± 0.03; *P* = .00006). The ratio of [^68^Ga]Ga-DOTA-Siglec-9 uptake between myocardial lesions and myocardium outside lesions was 2.7 ± 0.6. The lesion-to-blood ratio of [^68^Ga]Ga-DOTA-Siglec-9 uptake was 1.4 ± 0.5.

### Ex vivo autoradiography and biodistribution

Our autoradiography results revealed co-localization of [^68^Ga]Ga-DOTA-Siglec-9 uptake and myocardial lesions (Figure 4B). Autoradiography showed a 5.3 ± 1.2-fold higher uptake of [^68^Ga]Ga-DOTA-Siglec-9 in the myocardial lesions of immunized rats (n = 8) than in the myocardium outside lesions (PSL/mm^2^, 5.3 ± 1.0 *vs* 1.1 ± 0.4; *P* = .0002), and 5.5 ± 1.1-fold higher uptake than in the myocardium of control rats (PSL/mm^2^, 1.0 ± 0.1; n = 6; *P* = .00006; Figure 5B, C).

The highest radioactivity concentration in autoradiography was observed in the area where histologically there was edema, hemorrhage, and fibroblasts and scattered inflammatory cells. This area was almost devoid of cardiomyocytes. The number of inflammatory cells appeared to be higher in the surrounding myocardial tissue. The biodistribution of [^68^Ga]Ga-DOTA-Siglec-9 is presented in Table 1. In the immunized rats, [^68^Ga]Ga-DOTA-Siglec-9 accumulation was higher in the heart, thymus, and white adipose tissue than in the control rats. Positivity in autoradiography reflects, at least in part, relatively higher radioactivity in blood/plasma compared to myocardium.

**Table 1:** Ex vivo biodistribution of [^68^Ga]Ga-DOTA-Siglec-9 and [^68^Ga]Ga-DOTA-control peptides in rats at 70 minutes after intravenous injection

	[^68^Ga]Ga-DOTA-Siglec-9			[^68^Ga]Ga-DOTA-control	
	Immunized rats (n = 8)	Control rats (n = 6)	*P* value	Immunized rats (n = 4)	*P* value
Blood	0.23 ± 0.08	0.18 ± 0.05	.18	0.32 ± 0.02	.06
Heart	0.14 ± 0.04	0.07 ± 0.02	.007*	0.14 ± 0.03	.78
Intestine (without content)	0.20 ± 0.06	0.15 ± 0.07	.11	0.13 ± 0.02	.04*
Kidney	19.34 ± 9.12	14.37 ± 5.60	.22	6.69 ± 0.93	.007*
Liver	1.00 ± 0.62	0.75 ± 0.20	.14	0.18 ± 0.02	.004**
Lung	0.45 ± 0.18	0.32 ± 0.15	.18	0.23 ± 0.01	.01*
Lymph node	0.23 ± 0.09	0.18 ± 0.06	.18	0.17 ± 0.01	.04**
Muscle	0.06 ± 0.01	0.04 ± 0.02	.08	0.05 ± 0.004	.46
Plasma	0.44 ± 0.07	0.35 ± 0.12	.08	0.56 ± 0.05	.009*
Pancreas	0.11 ± 0.04	0.08 ± 0.02	.17	0.10 ± 0.02	.99
Spleen	0.51 ± 0.27	0.41 ± 0.14	.45	0.21 ± 0.05	.02*
Thymus	0.15 ± 0.05	0.04 ± 0.01	.002***	0.08 ± 0.06	.10
White adipose tissue	0.06 ± 0.02	0.03 ± 0.01	.05**	0.06 ± 0.01	.71

The results are expressed as standardized uptake values (SUVs; mean ± SD).

*A statistically significant difference based on Student’s *t*,test.

**A statistically significant difference based on Mann–Whitney *U* test.

***A statistically significant difference based on Kolmogorov–Smirnov test.

**Figure 6:**
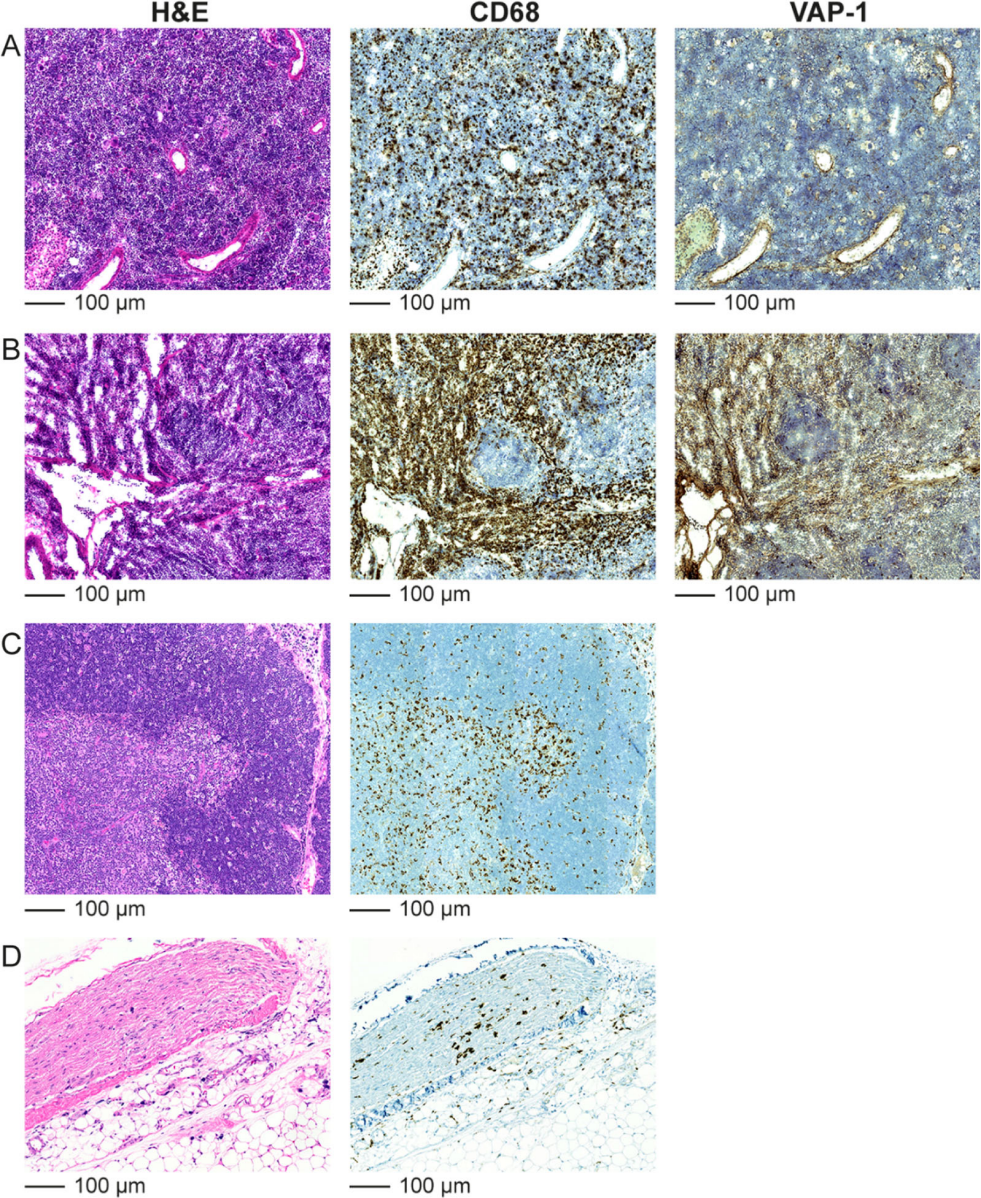
Hematoxylin and eosin (H&E), CD68 macrophage, and VAP-1 immunohistochemical staining of (**A**) spleen and (**B**) bone marrow cryosections from a rat with autoimmune myocarditis. Rat received intravenously anti-VAP-1 antibody 10 minutes before sacrifice and staining was performed using only the secondary antibody. H&E and CD68 macrophage staining of paraffin-embedded sections of (**C**) thymus and (**D**) white adipose tissue from a rat with autoimmune myocarditis

Immunohistochemical staining of spleen and bone marrow showed CD68-positive macrophages and VAP-1-positivity in these organs (Figure 6A, B). Immunohistochemical staining of thymus and white adipose tissue showed abundant CD68-positive macrophages (Figure 6C, D).

### Studies with [^68^Ga]Ga-DOTA-control peptide

In vivo studies with [^68^Ga]Ga-DOTA-control peptide showed 45% lower uptake ratio (myocardial lesions *vs* myocardium outside lesions) than [^68^Ga]Ga-DOTA-Siglec-9 (1.8 ± 0.5 *vs* 1.0 ± 0.05; *P* = .02). According to the ex vivo autoradiography assessment in immunized rats, [^68^Ga]Ga-DOTA-control peptide showed a 65% lower uptake ratio (myocardial lesions *vs* myocardium outside lesions) than [^68^Ga]Ga-DOTA-Siglec-9 (1.8 ± 0.2 *vs* 5.3 ± 1.2; *P* = .0006). The biodistribution results of [^68^Ga]Ga-DOTA-control peptide are presented in Table 1. [^68^Ga]Ga-DOTA-control peptide accumulation was significantly lower in the intestine, kidney, liver, lung, lymph node, and spleen and significantly higher in the plasma of the immunized rats compared to the accumulation of [^68^Ga]Ga-DOTA-Siglec-9.

### In vitro binding of [^68^Ga]Ga-DOTA-Siglec-9

The quantitative in vitro autoradiography analysis revealed significantly lower [^68^Ga]Ga-DOTA-Siglec-9 binding (49% reduction) in sections incubated with 100-fold molar excess of non-labeled DOTA-Siglec-9 peptide (blocked binding) than with the total [^68^Ga]Ga-DOTA-Siglec-9 binding (myocardial lesions *vs* myocardium outside lesions ratio 1.2 ± 0.1 *vs* 2.4 ± 0.2; *P* = .002), thus indicating that tracer binding was specific.

## Discussion

We demonstrated the expression of VAP-1 in rats with autoimmune myocarditis and patients with cardiac sarcoidosis. In vivo PET imaging of rats with VAP-1-targeted [^68^Ga]Ga-DOTA-Siglec-9 and ex vivo autoradiography showed uptake in the myocardial lesion area with modest lesion-to-background ratios.

The immunofluorescence staining showed that VAP-1 is expressed on vascular endothelial cells in myocardial lesions with dense macrophage infiltration, while CD31 staining showed a capillary network in the myocardial lesions. Furthermore, immunohistochemical staining of human cardiac sarcoid showed VAP-1 expression co-localized with vascular structures.

The rat model of autoimmune myocarditis used has similarities to human giant cell myocarditis and is a well-established experimental model.^29–31^ Myocarditis is believed to be initiated by macrophages and T-cell infiltration.^29^ Consistent with our previous findings,^6^ multiple focal lesions developed in the heart, and macrophages were the most prevalent cell type in this disease model.

Our in vivo PET results showed higher [^68^Ga]Ga-DOTA-Siglec-9 uptake in rats with myocarditis than in those without myocarditis, while autoradiography confirmed localization in the myocardial lesions. Compared with [^68^Ga]Ga-DOTA-Siglec-9 uptake, the in vivo PET/CT and ex vivo autoradiography assessment of [^68^Ga]Ga-DOTA-control peptide binding showed a 45% and 65% reduction in the target-to-background ratio, respectively. In vitro blocking study with non-labeled DOTA-Siglec-9 peptide showed 49% lower target-to-background ratio. These studies with a negative control peptide and in vitro blocking confirmed the specificity of the [^68^Ga]Ga-DOTA-Siglec-9 signal.

Our biodistribution results showed high [^68^Ga]Ga-DOTA-Siglec-9 accumulation in the heart, thymus, and white adipose tissue of the immunized rats compared with the control rats. The high tracer uptake in the thymus and white adipose tissue of the immunized rats is in the line with abundant CD68-positive cells in the organs. Furthermore, immunohistochemical staining of spleen and bone marrow of the immunized rats showed CD68-positive cells and VAP-1 expression in the organs. Although this is an interesting finding, further studies are needed to determine whether they may represent indirect signs of myocarditis.

Several non-invasive molecular imaging approaches have been studied for the detection of autoimmune myocarditis, including [^18^F]FOL,^6^ [^11^C]methionine,^7^ [^68^Ga]Ga-NOTA-MSA (mannosylated human serum albumin),^32^ 18-kDa translocator protein (TSPO)-targeting PET,^11^ and fluorine-19-based CMR;^33^ however, none of these used an adhesion molecule-targeting PET probe. On autoradiography, [^68^Ga]Ga-DOTA-Siglec-9 showed similar contrast between myocardial lesions and myocardium outside lesions (5.3 ± 1.2) to that previously described for other tracers such as [^18^F]FDG (3.4 ± 0.7), [^11^C]methionine (2.1 ± 0.2), and [^18^F]FOL (7.8 ± 1.4) when used in the same model.^6,7^

### Limitations

A limitation of our study is that we were unable to directly compare the uptake of [^68^Ga]Ga-DOTA-Siglec-9 and [^18^F]FDG in myocardial lesions because of physiological [^18^F]FDG uptake in the myocardium. Our results revealed that VAP-1 is expressed in a rat model of autoimmune myocarditis and human cardiac sarcoidosis. However, the role of VAP-1 in the pathogenesis of myocarditis and sarcoidosis remains unknown. Investigating uptake of VAP-1-targeted [^68^Ga]Ga-DOTA-Siglec-9 in response to therapy remains a future study topic. Another limitation of our study is the modest target-to-background ratio in the in vivo PET images. While the first-in-human study of ^68^Ga-DOTA-Siglec-9 has been performed,^19^ a clinical imaging study with [^68^Ga]Ga-DOTA-Siglec-9 in patients with myocarditis or cardiac sarcoidosis would be needed to evaluate the value of this approach.

## Conclusions

VAP-1 was present in the rat model of autoimmune myocarditis. The uptake of VAP-1-targeted [^68^Ga]Ga-DOTA-Siglec-9 was higher in myocardial lesions than in control animals and control tissue. [^68^Ga]Ga-DOTA-Siglec-9 PET/CT is a potential approach for the detection of inflammation in myocardium.

## New Knowledge Gained

PET imaging with a VAP-1-targeted radioligand, [^68^Ga]Ga-DOTA-Siglec-9, shows an increase in myocardial lesions in rats.

### Supplementary Information

Below is the link to the electronic supplementary material.

Supplementary file1 (DOCX 6699 KB)

Supplementary file2 (PPTX 1246 KB)

Supplementary file3 (M4A 2698 KB)

## Abbreviations

α-SMA: α-Smooth muscle actin
[^18^F]FDG: 2-Deoxy-2-[^18^F]fluoro-d-glucose
[^68^Ga]Ga-DOTA-Siglec-9: Gallium-68-labeled 1,4,7,10-tetraazacyclododecane-*N*,*N*′,*N*″,*N*‴-tetraacetic acid conjugated Siglec 9 motif containing peptide
H&E: Hematoxylin and eosin staining
LV: Left ventricle
PET/CT: Positron emission tomography/computed tomography
ROI: Region of interest
Siglec: Sialic acid-binding immunoglobulin-like lectin
SUV: Standardized uptake value
VAP-1: Vascular adhesion protein-1; also called amine oxidase copper containing 3 (AOC3)
