# Supplementary material for: Vascular adhesion protein-1-targeted PET imaging in autoimmune myocarditis
Source: J Nucl Cardiol. 2023 Sep 27;30(6):2760–72. doi: 10.1007/s12350-023-03371-8 (PMC10682147; doi:10.1007/s12350-023-03371-8)
Supplement: Supplementary file 1 — Supplementary file1 (DOCX 6699 KB) [file 12350_2023_3371_MOESM1_ESM.docx]

**Supplemental material**


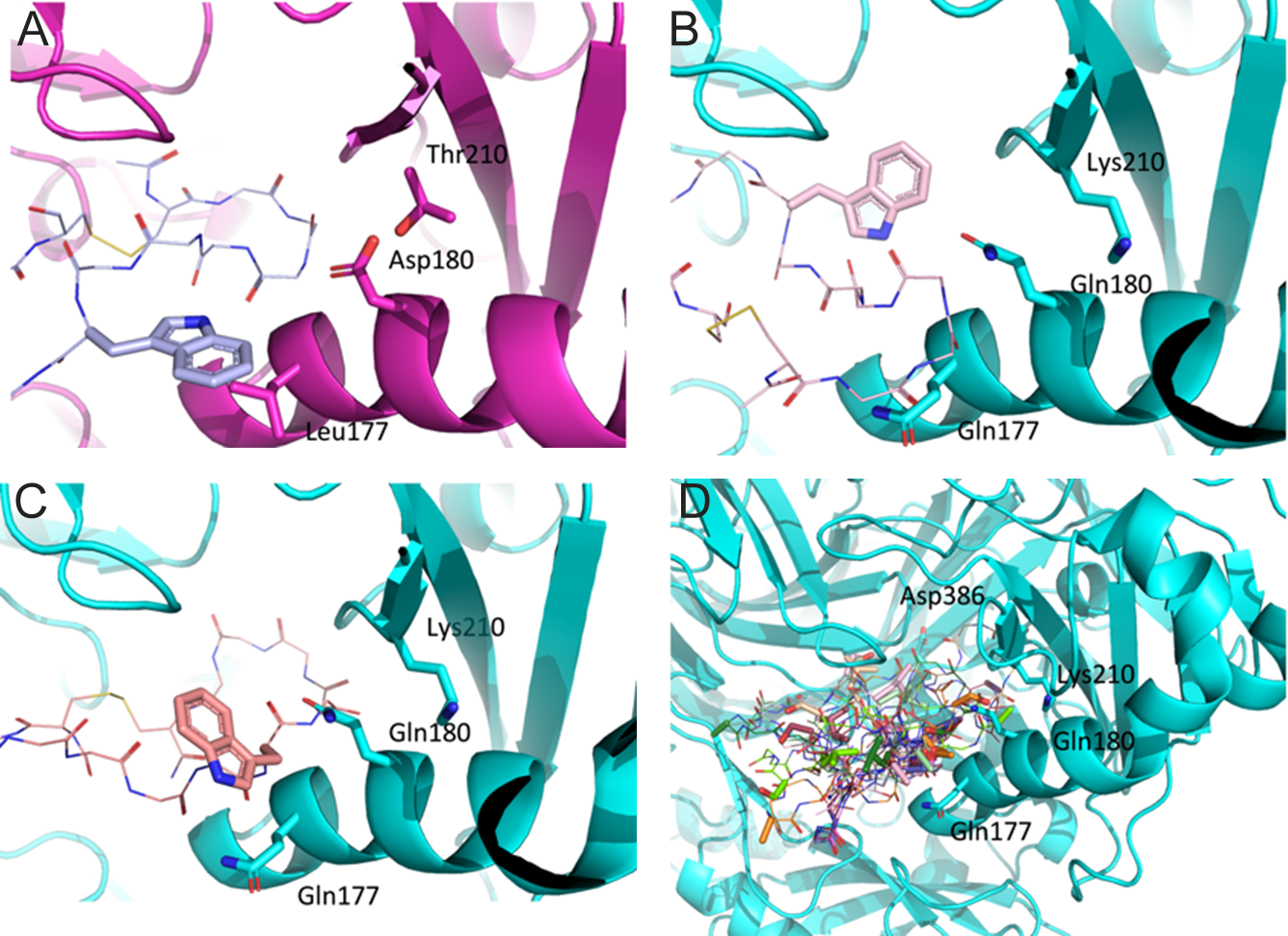


**Supplemental Figure 1**. Position of Trp8 (in sticks) in VAP-1 proteins in the specificity pocket (Leu177, Asp180, Thr210 in hVAP-1 and Gln177, Gln180, Lys210 in rVAP-1). (**A**) Trp8 binding site in the [^68^Ga]Ga-DOTA-Siglec-9 docked in hVAP-1 and (**B**) in rVAP-1. (**C**) Trp8 in the R3A/R9A peptide docked in rVAP-1. (**D**) [^68^Ga]Ga-DOTA-control peptide docked in rVAP-1 without Trp and Arg residues does not show any consistent binding mode.

**
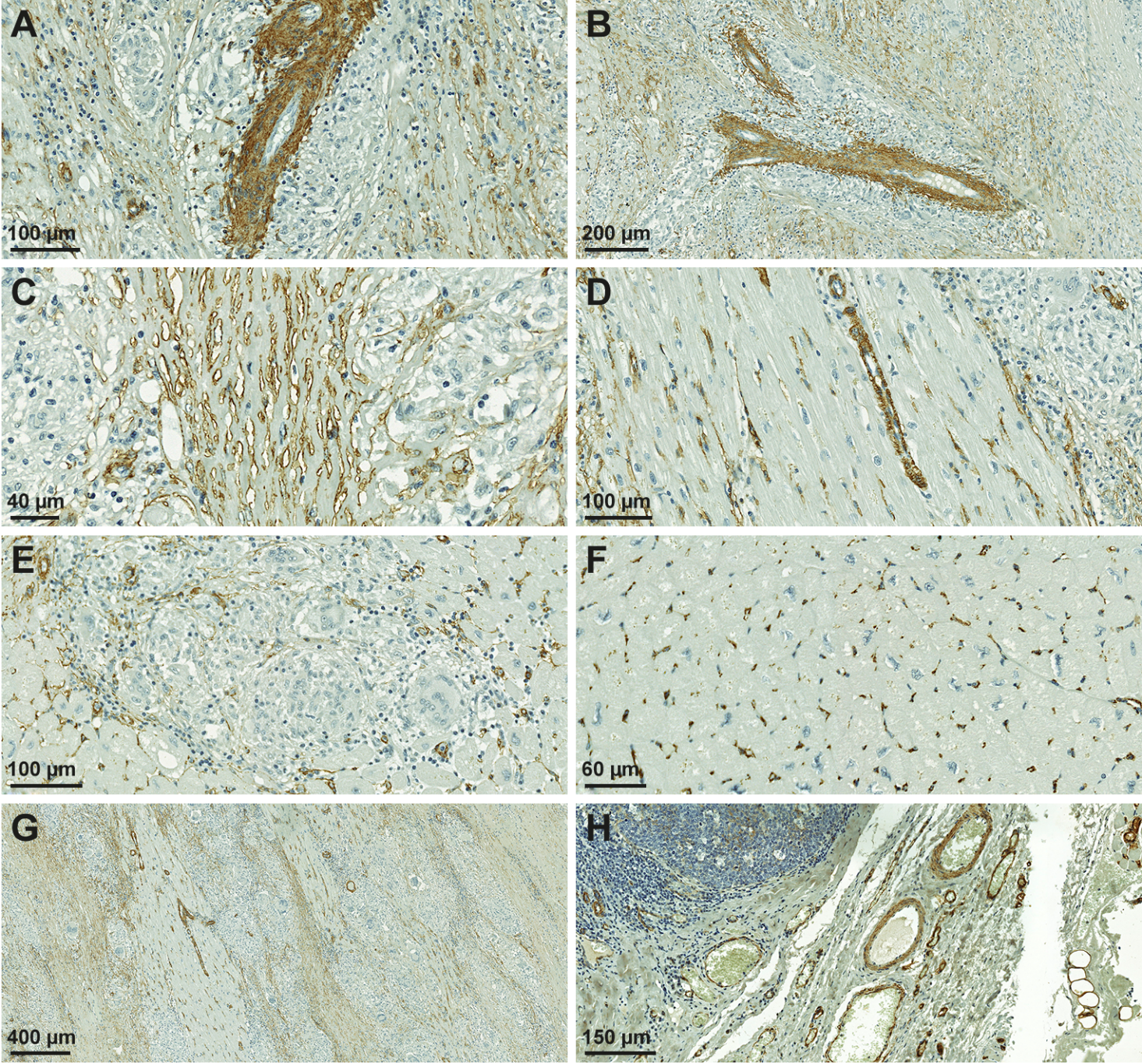
**

**Supplemental Figure 2.** VAP-1 staining in human tissues. VAP-1 staining positivity (brown) is observed in vascular smooth muscle cells (**A, B**) and in capillary endothelial cells (**A** – **H**). Cardiac sarcoidosis in (**A** – **G**). Region of maturing granulation tissue between sarcoid granulomas shows dense VAP-1- positive capillary network in (**c**). Inflammatory cells in sarcoid granulomas (**A** – **E**, and **G**) are VAP-1-negative. Capillary endothelial cells are VAP-1-positive in regions of normal myocardium (**F**). Inflammatory cells are VAP-1-negative also in tonsil (**H**), but vascular smooth muscle cells and adipocytes are positive in peritonsillar soft tissue.
