# Supplementary material for: Vascular adhesion protein-1-targeted PET imaging in autoimmune myocarditis
Source: J Nucl Cardiol. 2023 Sep 27;30(6):2760–72. doi: 10.1007/s12350-023-03371-8 (PMC10682147; doi:10.1007/s12350-023-03371-8)
Supplement: Supplementary file 2 — Supplementary file2 (PPTX 1246 KB) [file 12350_2023_3371_MOESM2_ESM.pptx]

## Slide 1
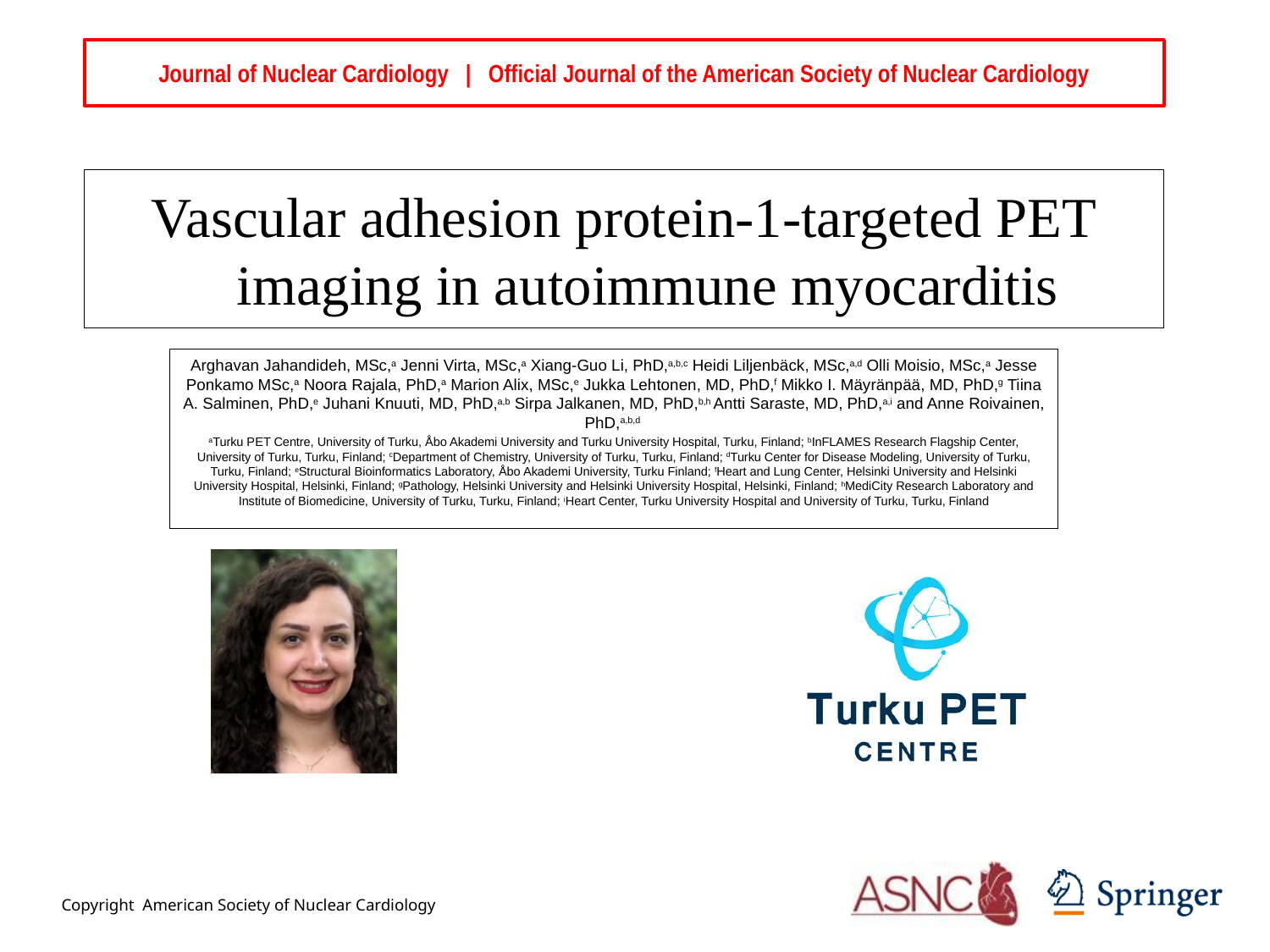

Journal of Nuclear Cardiology | Official Journal of the American Society of Nuclear Cardiology
# Vascular adhesion protein-1-targeted PET imaging in autoimmune myocarditis
Arghavan Jahandideh, MSc,a Jenni Virta, MSc,a Xiang-Guo Li, PhD,a,b,c Heidi Liljenbäck, MSc,a,d Olli Moisio, MSc,a Jesse Ponkamo MSc,a Noora Rajala, PhD,a Marion Alix, MSc,e Jukka Lehtonen, MD, PhD,f Mikko I. Mäyränpää, MD, PhD,g Tiina A. Salminen, PhD,e Juhani Knuuti, MD, PhD,a,b Sirpa Jalkanen, MD, PhD,b,h Antti Saraste, MD, PhD,a,i and Anne Roivainen, PhD,a,b,d
aTurku PET Centre, University of Turku, Åbo Akademi University and Turku University Hospital, Turku, Finland; bInFLAMES Research Flagship Center, University of Turku, Turku, Finland; cDepartment of Chemistry, University of Turku, Turku, Finland; dTurku Center for Disease Modeling, University of Turku, Turku, Finland; eStructural Bioinformatics Laboratory, Åbo Akademi University, Turku Finland; fHeart and Lung Center, Helsinki University and Helsinki University Hospital, Helsinki, Finland; gPathology, Helsinki University and Helsinki University Hospital, Helsinki, Finland; hMediCity Research Laboratory and Institute of Biomedicine, University of Turku, Turku, Finland; iHeart Center, Turku University Hospital and University of Turku, Turku, Finland
Copyright American Society of Nuclear Cardiology

## Slide 2
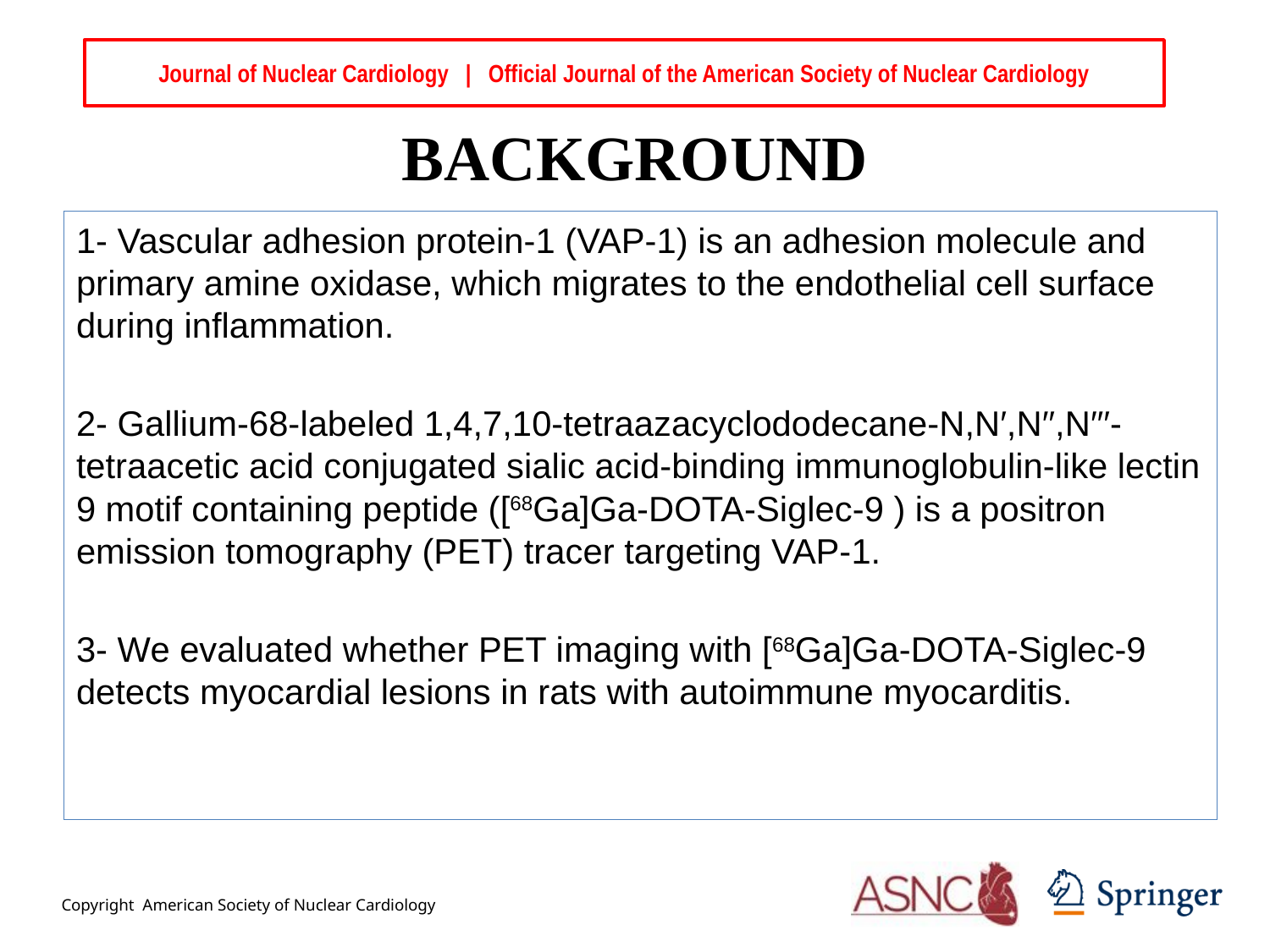

Journal of Nuclear Cardiology | Official Journal of the American Society of Nuclear Cardiology
# BACKGROUND
1- Vascular adhesion protein-1 (VAP-1) is an adhesion molecule and primary amine oxidase, which migrates to the endothelial cell surface during inflammation.
2- Gallium-68-labeled 1,4,7,10-tetraazacyclododecane-N,N′,N′′,N′′′-tetraacetic acid conjugated sialic acid-binding immunoglobulin-like lectin 9 motif containing peptide ([68Ga]Ga-DOTA-Siglec-9 ) is a positron emission tomography (PET) tracer targeting VAP-1.
3- We evaluated whether PET imaging with [68Ga]Ga-DOTA-Siglec-9 detects myocardial lesions in rats with autoimmune myocarditis.
Copyright American Society of Nuclear Cardiology

## Slide 3
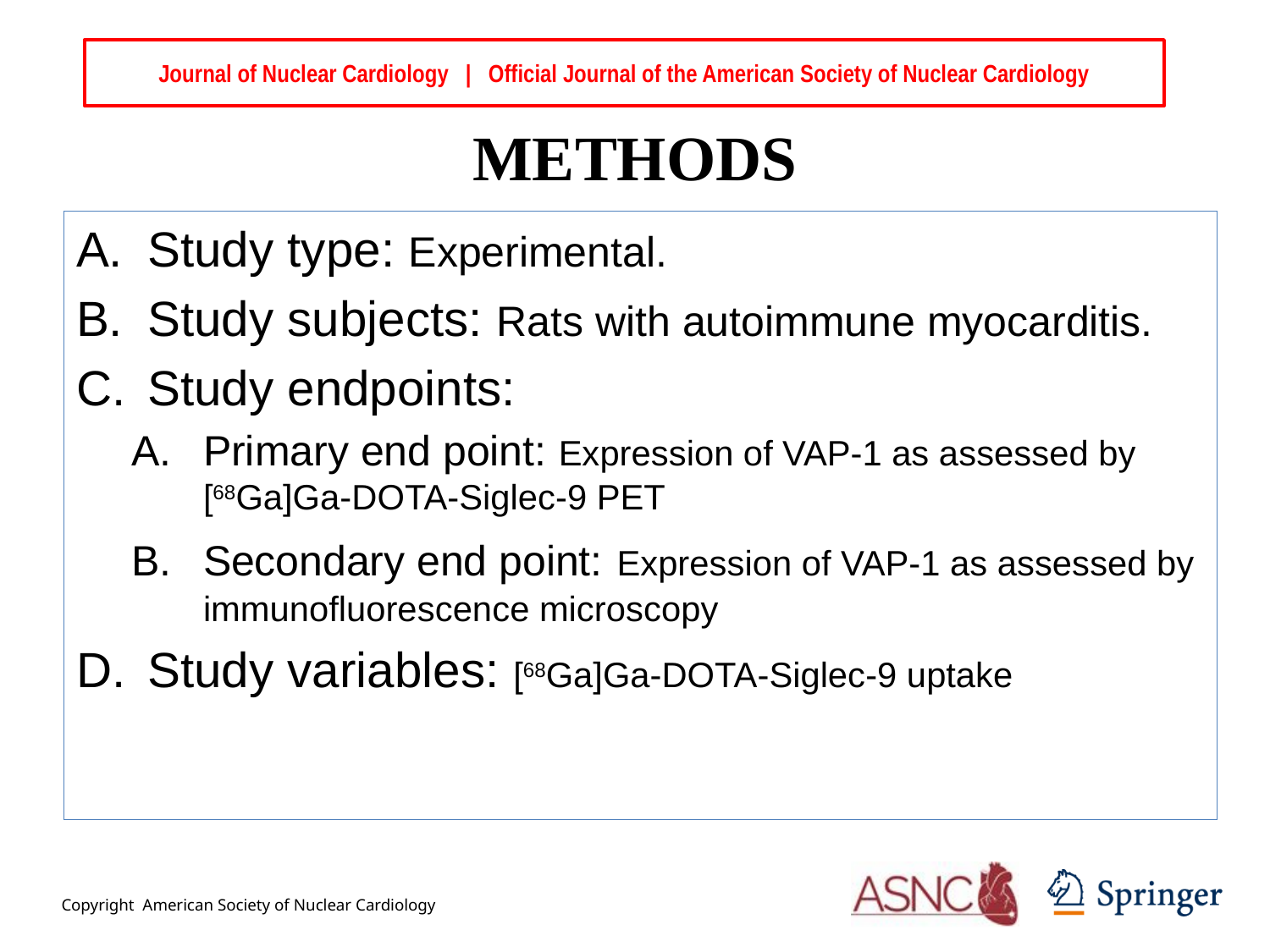

Journal of Nuclear Cardiology | Official Journal of the American Society of Nuclear Cardiology
# METHODS
Study type: Experimental.
Study subjects: Rats with autoimmune myocarditis.
Study endpoints:
Primary end point: Expression of VAP-1 as assessed by [68Ga]Ga-DOTA-Siglec-9 PET
Secondary end point: Expression of VAP-1 as assessed by immunofluorescence microscopy
Study variables: [68Ga]Ga-DOTA-Siglec-9 uptake
Copyright American Society of Nuclear Cardiology

## Slide 4
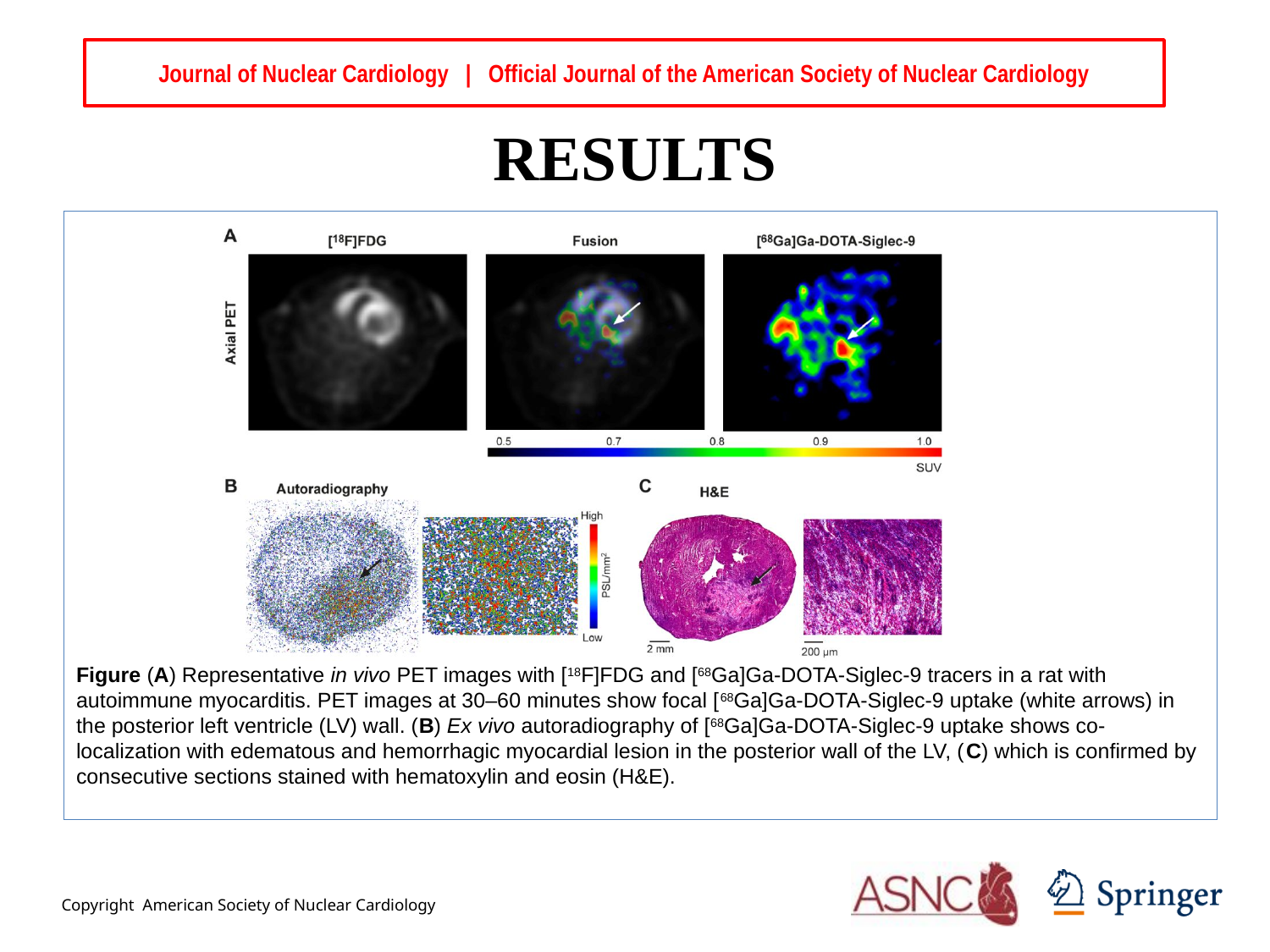

Journal of Nuclear Cardiology | Official Journal of the American Society of Nuclear Cardiology
# RESULTS
Figure (A) Representative in vivo PET images with [18F]FDG and [68Ga]Ga-DOTA-Siglec-9 tracers in a rat with autoimmune myocarditis. PET images at 30–60 minutes show focal [68Ga]Ga-DOTA-Siglec-9 uptake (white arrows) in the posterior left ventricle (LV) wall. (B) Ex vivo autoradiography of [68Ga]Ga-DOTA-Siglec-9 uptake shows co-localization with edematous and hemorrhagic myocardial lesion in the posterior wall of the LV, (C) which is confirmed by consecutive sections stained with hematoxylin and eosin (H&E).
Copyright American Society of Nuclear Cardiology

## Slide 5
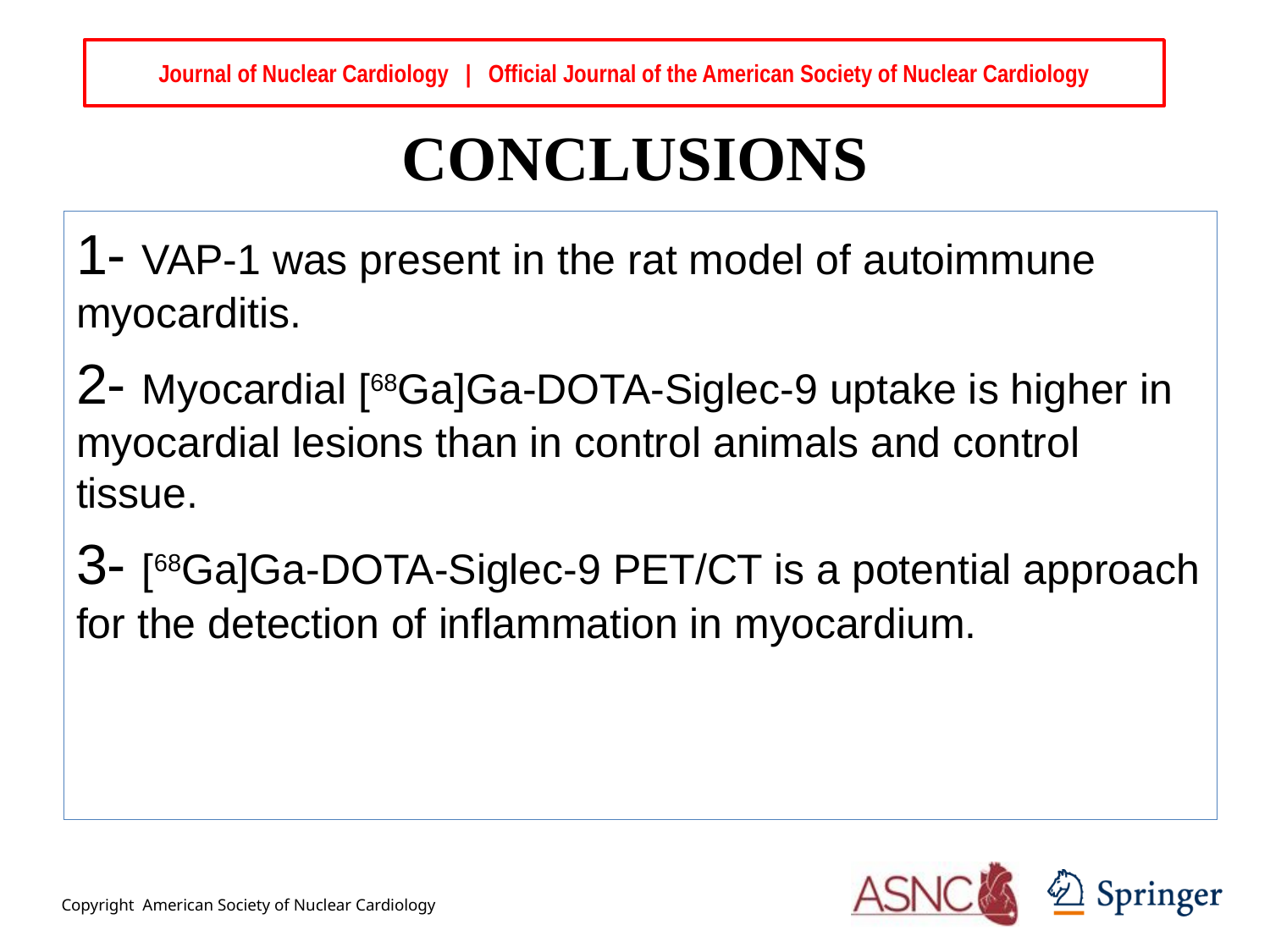

Journal of Nuclear Cardiology | Official Journal of the American Society of Nuclear Cardiology
# CONCLUSIONS
1- VAP-1 was present in the rat model of autoimmune myocarditis.
2- Myocardial [68Ga]Ga-DOTA-Siglec-9 uptake is higher in myocardial lesions than in control animals and control tissue.
3- [68Ga]Ga-DOTA-Siglec-9 PET/CT is a potential approach for the detection of inflammation in myocardium.
Copyright American Society of Nuclear Cardiology
